# The bivalve* Thyasira* cf.* gouldi* hosts chemoautotrophic symbiont populations with strain level diversity

**DOI:** 10.7717/peerj.3597

**Published:** 2017-07-26

**Authors:** Bonita McCuaig, France Liboiron, Suzanne C. Dufour

**Affiliations:** Department of Biology, Memorial University of Newfoundland, St John’s, NL, Canada

**Keywords:** Thyasiridae, 16S rRNA, Phylogeny, Chemosymbiosis, Rubisco

## Abstract

Invertebrates from various marine habitats form nutritional symbioses with chemosynthetic bacteria. In chemosynthetic symbioses, both the mode of symbiont transmission and the site of bacterial housing can affect the composition of the symbiont population. Vertically transmitted symbionts, as well as those hosted intracellularly, are more likely to form clonal populations within their host. Conversely, symbiont populations that are environmentally acquired and extracellular may be more likely to be heterogeneous/mixed within host individuals, as observed in some mytilid bivalves. The symbionts of thyasirid bivalves are also extracellular, but limited 16S rRNA sequencing data suggest that thyasirid individuals contain uniform symbiont populations. In a recent study, *Thyasira* cf. *gouldi* individuals from Bonne Bay, Newfoundland, Canada were found to host one of three 16S rRNA phylotypes of sulfur-oxidizing gammaproteobacteria, suggesting environmental acquisition of symbionts and some degree of site-specificity. Here, we use Sanger sequencing of both 16S RNA and the more variable ribulose-1,5-bisphosphate carboxylase (RuBisCO) PCR products to further examine *Thyasira* cf. *gouldi* symbiont diversity at the scale of host individuals, as well as to elucidate any temporal or spatial patterns in symbiont diversity within Bonne Bay, and relationships with host OTU or size. We obtained symbiont 16S rRNA and RuBisCO Form II sequences from 54 and 50 host individuals, respectively, during nine sampling trips to three locations over four years. Analyses uncovered the same three closely related 16S rRNA phylotypes obtained previously, as well as three divergent RuBisCO phylotypes; these were found in various pair combinations within host individuals, suggesting incidents of horizontal gene transfer during symbiont evolution. While we found no temporal patterns in phylotype distribution or relationships with host OTU or size, some spatial effects were noted, with some phylotypes only found within particular sampling sites. The sequencing also revealed symbiont populations within individual hosts that appeared to be a mixture of different phylotypes, based on multiple base callings at divergent sites. This work provides further evidence that *Thyasira* cf. *gouldi* acquires its symbionts from the environment, and supports the theory that hosts can harbour symbiont populations consisting of multiple, closely related bacterial phylotypes.

## Introduction

Symbioses between animals and bacteria are ubiquitous and, in many cases, advantageous to the host (McFall-Ngai et al., 2013). Animals often benefit from symbiont-derived metabolic products, and in a class of animal-bacteria relationships called chemosynthetic symbioses, marine invertebrates receive nutrients from chemoautotrophic bacterial symbionts. Since the discovery of chemosynthetic symbioses in giant tubeworms from hydrothermal vents (Felbeck, 1981; Jones, 1981), invertebrates from various phyla and marine habitats were found to establish nutritional symbioses with a wide diversity of chemoautotrophic bacteria (Cavanaugh et al., 2006; Dubilier, Bergin & Lott, 2008; Petersen et al., 2012). The degree of symbiont specificity varies among host species: some harbour clonal populations, while others can form symbioses with more than one bacterial strain, in either single or mixed populations (Duperron et al., 2008a; Petersen et al., 2012; Brissac et al., 2016). As mixed symbiont populations may confer hosts greater metabolic flexibility (Ikuta et al., 2016), examining symbiont specificity can inform us on how hosts might respond to environmental change, and how symbioses evolve and break down (Sachs, Skophammer & Regus, 2011).

Among the factors that can influence symbiont specificity, the mode of symbiont transmission (vertical or environmental) has received much attention. Hosts that transmit symbionts vertically (usually within the eggs) tend to form highly specific relationships with symbionts, which often form clonal populations (Goffredi et al., 2003; Wernegreen, 2005; Caro et al., 2007; Bright & Bulgheresi, 2010). Other host species, such as those that obtain symbionts environmentally from free-living bacterial populations (Nussbaumer, Fisher & Bright, 2006; Won, Jones & Vrijenhoek, 2008; Bright & Bulgheresi, 2010; Vrijenhoek, 2010), may be less specific and harbour mixed symbiont populations (Vrijenhoek, Duhaime & Jones, 2007; Moran, McCutcheon & Nakabachi, 2008; Ikuta et al., 2016). Less well understood is the importance of cellular integration on symbiont specificity, especially in hosts that are colonized by free-living bacteria. In many invertebrates (e.g., vesicomyid bivalves and giant tubeworms; Cavanaugh et al., 2006) symbionts are maintained within host cells whereas in others, symbionts are internalized but extracellular (e.g., thyasirid and some bathymodiolin bivalves with symbionts held among microvilli of gill epithelial cells; Dufour, 2005; Duperron et al., 2008b), or are epibiotic, attached to the external surface of the body (e.g., nematodes; Ott et al., 1991). Among hosts with environmentally acquired symbionts, those that house symbionts intracellularly might show more specificity than those with external symbionts, as internalization processes can be selective (Brissac et al., 2016). For example, lucinid bivalves and vestimentiferan tubeworms are colonized by symbionts as juveniles, and contain one or two, metabolically divergent, locally sourced intracellular symbiont phylotype(s) as adults (Zimmerman et al., 2014; Brissac et al., 2016). In contrast, hosts with extracellular symbionts may associate with a broader, more variable range of symbiont types, as observed in wood-fall mussels having mixed populations of 5–6 divergent phylotypes (Duperron et al., 2008a). However, a different situation has been observed in thyasirid bivalves with extracellular symbionts: *Thyasira* cf. *gouldi* conspecifics from the same fjord (Bonne Bay, Newfoundland, Canada) associated with one of three highly similar 16S rRNA phylotypes of sulfur-oxidizing gammaproteobacteria (Batstone & Dufour, 2016). These observations highlight the fact that the mechanisms of symbiont selection in extracellular symbioses are not known, and may differ markedly amongst host taxa.

Thyasirids can be abundant in organically enriched coastal waters such as fjords, and are a valuable group in which to study extracellular chemosynthetic symbioses. In Bonne Bay, *Thyasira* cf. *gouldi* form a complex of three distinct operational taxonomic units (OTUs), identified through 18S rRNA, 23S rRNA, and CO1 sequencing (Batstone et al., 2014). OTUs 1 and 2 have elongated gill filaments housing thioautotrophic bacteria, while OTU 3 has shorter gill filaments and is asymbiotic (Batstone et al., 2014). Symbiotic and asymbiotic OTUs of *T.* cf. *gouldi* create elaborate burrows within the sediment using their extensible foot (Zanzerl & Dufour, 2017). In symbiotic thyasirids, burrow formation has been interpreted as a mechanism to “mine” for the sulfur compounds the symbiont requires (Dufour & Felbeck, 2003; Dando, Southward & Southward, 2004), while in some asymbiotic thyasirids, burrows have been associated with pedal feeding, a type of deposit feeding where particles are collected by the foot (Zanzerl & Dufour, 2017). Thyasirid bioirrigation leads to the establishment of oxic/anoxic interfaces around burrow linings (Dando, Southward & Southward, 2004; Hakonen, Hulth & Dufour, 2010) and likely favours colonization of sulfur-oxidizing bacteria. The presence of magnetosome particles in thyasirid symbionts suggests that, in their free-living state, symbionts navigate to burrow linings, where hosts can collect them on the mucociliary surface of their extensile foot and bring them in contact with their gills (Dufour et al., 2014). This proposed environmental mode of symbiont uptake likely explains why different thyasirid species associate with symbionts belonging to different phylogenetic groups (Rodrigues & Duperron, 2011; Batstone & Dufour, 2016).

Here, we examine the symbiont populations of *Thyasira* cf. *gouldi* from Bonne Bay (Canada) in greater detail by sequencing fragments of both the 16S rRNA gene and the ribulose-1,5-bisphosphate carboxylase (RuBisCO) gene. The latter gene was chosen because it evolves more rapidly than the 16S rRNA gene, and has been phylogenetically informative in other studies of chemoautotrophic symbionts (Blazejak et al., 2006; Vrijenhoek, Duhaime & Jones, 2007). Hence, the dual-marker approach can provide us with a better understanding of extracellular symbiont population diversity and site-specific adaptation in thyasirids, and inform us on the potential for gene exchange between symbionts and free-living bacteria in surrounding sediments. We examine: (1) the relationship between16S rRNA phylotype, RuBisCO phylotype, and host OTU; (2) site specificity of phylotypes at three Bonne Bay sampling locations; (3) temporal patterns in phylotype presence; and (4) relationships between gene phylotype and host size.

## Materials & Methods

### Sample collection

**Figure 1:**
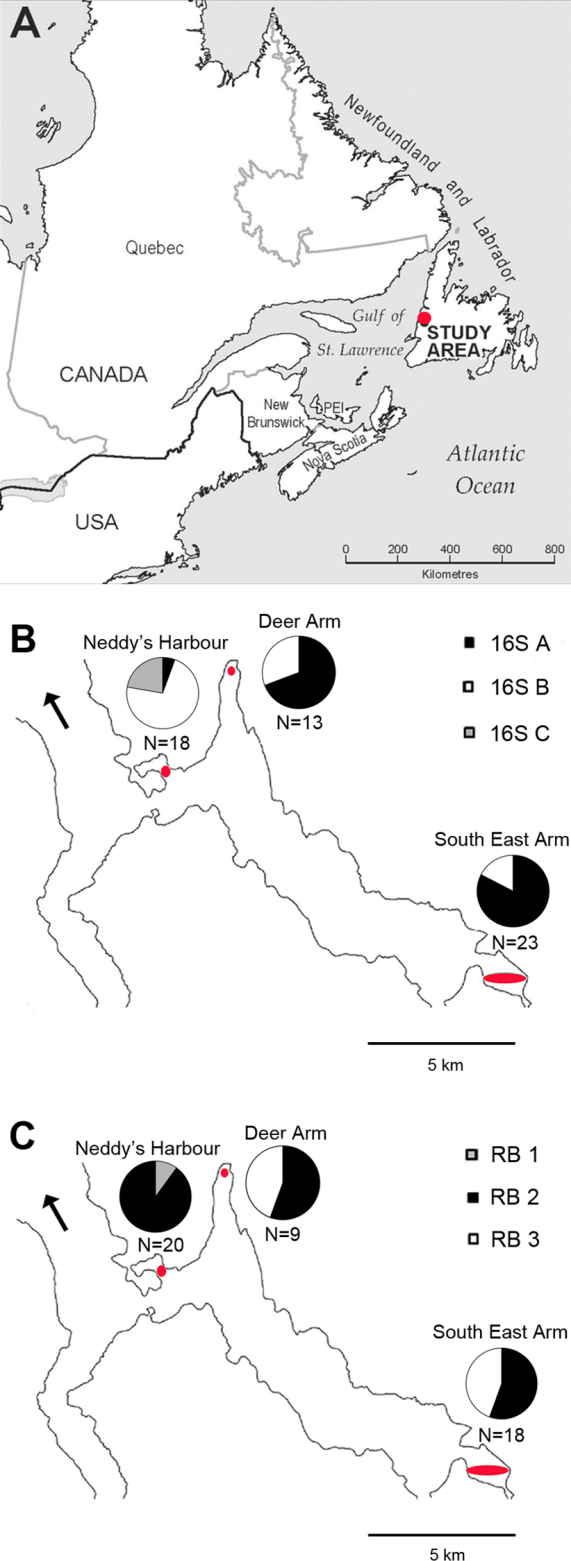
Map of phylotype distributions within Bonne Bay. (A) The location of the study area within Eastern Canada; (B) distribution of 16S rRNA phylotypes; (C) distribution of RuBisCO phylotypes. Sampling sites are located within the red ovals. N indicates the number of host specimens in which sequences were obtained from each site. The black arrow points to the Gulf of St Lawrence (mouth of Bonne Bay).

Thyasirids were collected from Bonne Bay, Newfoundland, Canada on nine occasions between October 2009 and May 2012 (Table S1); permits for field sampling (NL 572 11 and NL 992 12) were obtained from Fisheries and Oceans Canada. Sediment was collected using a Peterson grab (radius = 10.5 cm, length = 30 cm, volume = 0.01 m^3^) from three sites within the fjord (Fig. 1): Neddy’s Harbour (17–30 m depth), Deer Arm (29–36 m depth) and South East Arm (20–35 m depth). Thyasirids were retrieved from sediments using a sieve with 1 mm mesh, and symbiotic individuals (*Thyasira* cf. *gouldi* OTU 1 and 2, distinguished by their shell shape; Batstone et al., 2014), were retained.

### DNA extraction and gene sequencing

The gills of symbiotic *Thyasira* cf. *gouldi* specimens were dissected and immediately frozen or stored in 95% ethanol. Following the protocol for animal tissues, total DNA was extracted from gills using QIAGEN DNeasy^®^ Blood and Tissue kit spin columns and stored at −20 °C in the elusion buffer provided. The PCR amplification of 16S rRNA and RuBisCO gene sequences was conducted using 12.5 µl of Green Dream Master Mix, 1.5 µl of template DNA, 1 µl of forward primer, 1 µl of reverse primer, and 9 µl of water. A 1323 bp fragment of the 16S rRNA gene was amplified using primers 27F (5′AGAGTTTGGATCMTGGCTCAG 3′) and 1492R (5′CGGTTACCTTGTTACGACTT 3′) (Lane, 1991). Thermocycler settings were: 94 °C for 3 min, 35 cycles of (94 °C for 1 min, 50 °C for 30 sec, 72 °C for 1.5 min) and a final extension of 72 °C for 10 min. A 296 bp fragment of the RuBisCO Form II gene (previously identified within the symbionts of *T.* cf. *gouldi*; Dufour et al., 2014) was amplified using primers 663F (5′ATCATCAARCTSGGCCTGCGTCCC 3′) and 1033R (5′MGAGGTGACSGCRCCGTGRCCRGCMCRTG 3′) (Widmer et al., 1998); initial denaturation was at 95 °C for 2 min, followed by 30 cycles of 95 °C for 1 min, 62 °C for 1 min and 72 °C for 30 sec, and a final elongation at 72 °C for 5 min. PCR products were cleaned using Agencourt AMPure XP Beads (Beckman Coulter, Brea, CA, USA) following the manufacturer’s protocol, and sent to The Center for Advanced Genomics, Toronto, Canada for Sanger sequencing.

### Phylogenetic analysis

Sequences were checked for quality, manually trimmed from both ends, and corresponding forward and reverse sequences from a single clam individual were combined into contiguous sequences (contigs) using SEQUENCHER^®^ 5.1 (Gene Codes Corp., Ann Arbor, MI, USA).

Contigs were then aligned in MEGA 7 (Kumar, Stecher & Tamura, 2016) using the ClustalW algorithm (Thompson, Higgins & Gibson, 1994). We paid particular attention to any sites with double peaks in the chromatographs (i.e., where IUPAC degenerate base symbols were assigned by the sequencing software) on corresponding forward and reverse sequences: see Fig. S1 for examples. We examined the identity and position of double bases within the alignment, as they could signal the presence of more than one strain or phylotype within a particular host bivalve (as in the vesicomyid tubeworms examined by Vrijenhoek, Duhaime & Jones, 2007).

Maximum likelihood trees were constructed for both 16S rRNA and RuBisCO genes obtained herein, with the tree for the former gene including additional sequences from Batstone & Dufour (2016). Sequences with degenerate bases were not included in the RuBisCO tree as the high number of heterogeneous sequences considerably reduced bootstrap numbers. Appropriate models were identified using MEGA7. Three distinct clusters were identified in each tree, and representative sequences of the highest quality (and without ambiguities) were selected for comparison to sequences of chemosynthetic symbionts and free-living bacteria for which both 16S rRNA and RuBisCO Form II sequences were available in Genbank (Table S2).

The degree of divergence within and between phylotype groups was calculated in MEGA 7, using the Jukes-Cantor model for 16S rRNA and the Tamura-Nei model (Tamura & Nei, 1993) with 5 discrete gamma distributions for RuBisCO. Variance from the model was estimated with 1000 bootstrap replicates. Percent similarity between pairs of representative sequences from each phylotype was calculated using BLAST.

### Statistical analysis of gene pairings and of spatial, temporal, and host size effects

We used chi-square tests to examine whether 16S rRNA and RuBisCO phylotypes identified unambiguously from a series of host specimens were independent of each other. A similar approach was used to assess independence between 16S and RuBisCO phylotypes and (1) sampling site, (2) sampling date, and (3) host size (set as the following categories: <3 mm, 3–4 mm, and >4 mm). Statistical analyses were performed using R (Ihaka & Gentleman, 1996).

## Results

### Description of sequences and evolutionary patterns

From the bivalves examined in this study, we obtained 16S rRNA and RuBisCO Form II sequences from 54 and 50 host individuals, respectively (Table S1). A phylogenetic tree using all sequences from this study was constructed for each gene; only sequences with no degenerate bases were used for the RuBisCO tree (Figs. S2 and S3). The 16S rRNA sequences formed 3 phylotypes, in agreement with (Batstone & Dufour, 2016); these are hereafter referred to as 16S A, 16S B, and 16S C. RuBisCO sequences varied but could also be grouped into three phylotypes: RB 1, RB 2 and RB 3.

**Figure 2:**
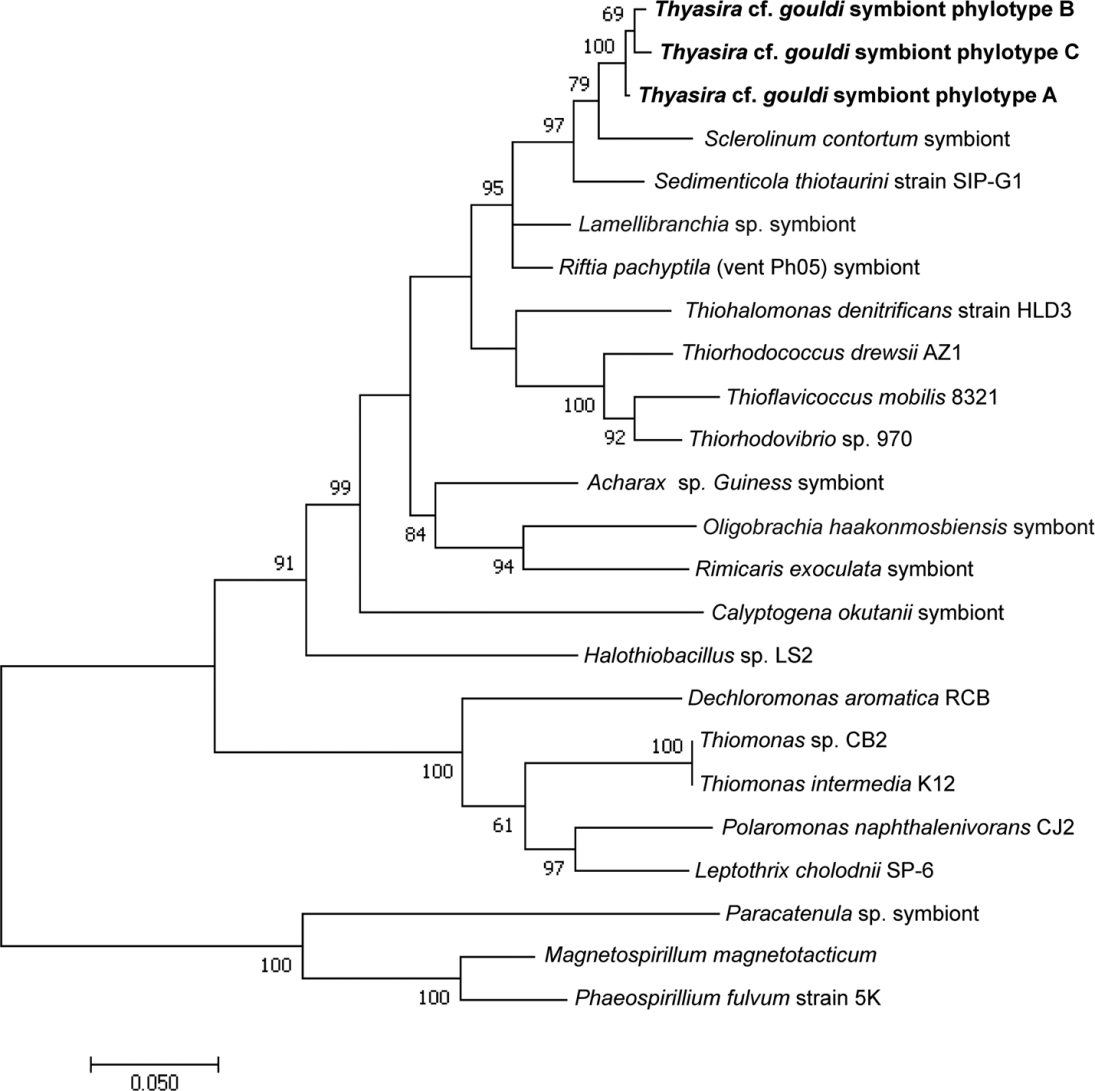
16S rRNA Maximum Likelihood phylogenetic tree. This tree was created using the General Time Reversible Model (Nei & Kumar, 2000) in MEGA7 (Kumar, Stecher & Tamura, 2016). The log likelihood of this tree was −8456.70. A discrete Gamma distribution of 5 was used to model evolutionary rate differences among sites, and the model allowed for some sites to be evolutionarily invariable ([I +] 31.81% sites). A total of 1,281 positions were used in the final dataset. 1,000 bootstrap replicates were conducted, and bootstrap values >50 are shown. The scale bar represents the number of nucleotide substitutions per site. Accession numbers are in Table S1.

**Figure 3:**
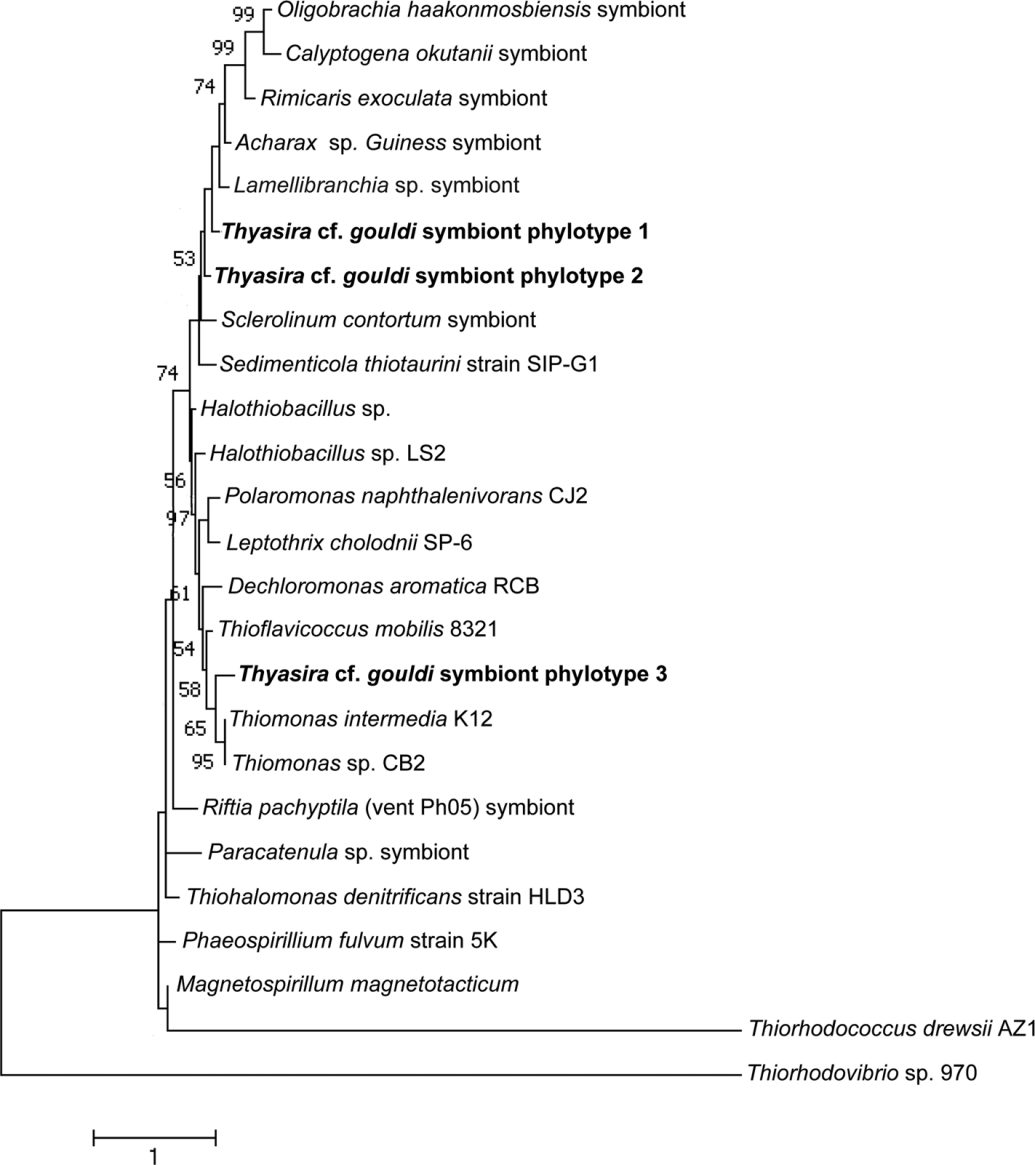
RuBisCO phylogenetic tree. This maximum likelihood tree was created using the Tamura-Nei model (Tamura & Nei, 1993). The log likelihood of this tree was −3880.57. A discrete Gamma distribution of 5 was used to model evolutionary rate differences among sites. A total of 284 positions were used in the final dataset. 1,000 bootstrap replicates were conducted, and bootstrap values >50 are shown. MEGA7 was used for alignment of sequences and tree construction (Kumar, Stecher & Tamura, 2016). The scale bar indicates the number of nucleotide substitutions per site. Accession numbers are in Table S1.

For each gene, evolutionary distances between and within phylotypes were calculated (Table 1). Although there was clear sequence variation within phylotypes (Table S3), no measurable evolutionary distances were observed within phylotypes of either gene, likely because most discrepancies were within wobble positions. Evolutionary distances between phylotypes were greater for the RuBisCO gene than for 16S rRNA. 16S A and 16S B are more similar to each other (evolutionary distance = 0.002) than to 16S C (evolutionary distances = 0.008 and 0.007, respectively). A similar pattern is seen among RuBisCO sequences, with RB 3 being the most distant, as we calculated distance values of 0.229 and 0.267 in comparison to RB 1 and RB 2, respectively. A clear divergence in the evolutionary history of 16S rRNA and RuBisCO genes within the *Thyasira* cf. *gouldi* symbionts was evident upon examination of phylogenetic trees that included sequences from the same free-living bacteria and chemosynthetic symbionts (Figs. 2 and 3).

**Table 1:** Evolutionary Distance matrices for 16S rRNA and RuBisCO phylotypes. The number of sequences (specimens) for each phylotype is in parentheses. The percent similarity between pairs of representative sequences is given in italics.

**16S rRNA**^a^	**16S A** (29)	**16S B** (21)
**16S B** (21)	0.002 ± 0.001; *99%*	
**16S C** (4)	0.008 ± 0.002; *99%*	0.007 ± 0.002; *99%*
**RuBisCO**^b^	**RB 1** (2)	**RB 2** (33)
**RB 2** (33)	0.131 ± 0.024; *87%*	
**RB 3** (12)	0.229 ± 0.023; *80%*	0.267 ± 0.041; *77%*

**Notes.**

a Calculated using the Jukes-Cantor model with 1,000 bootstrap replicates.

b Calculated using the Tamura-Nei model and 5 gamma distributions with 1,000 bootstrap replicates.

### Evidence for multiple symbiont phylotypes within a host

Sequences with degenerate bases were commonly observed (Table S3). Of the 54 16S rRNA sequences, 11 had a single instance of a high-quality call of multiple bases, while 11 others possessed between three and 12 degenerate bases in variable gene regions. Based on alignments, sequences with degenerate bases suggested a combination of slightly varying sequences within a single phylotype; in no cases did there appear to be more than one 16S rRNA phylotype within a single host bivalve.

Among the 50 RuBisCO sequences, 29 showed no ambiguities, while 18 showed high quality calls of multiple bases at sites varying within a given symbiont phylotype (i.e., the host appeared to contain multiple strains within a particular RB phylotype). Three individuals showed multiple calls at sites that suggested a mixture of symbiont phylotypes RB 2 and RB 3 (Fig. S3).

### Phylotypes and associated host or symbiont characteristics

We obtained corresponding, unambiguously categorized 16S rRNA and RuBisCO sequences from 33 host specimens and noted patterns in gene pairings within host individuals (Table 2). Phylotypes 16S C and RB 1 appeared associated with each other: two of the four host specimens with 16S C had RB 1 (no RuBisCO phylotype data could be obtained from the remaining two host specimens due to insufficient volumes of extracted DNA). Similarly, all 15 hosts containing the 16S B phylotype possessed RB 2. In contrast, six of the 16 *Thyasira* cf. *gouldi* individuals with symbiont phylotype 16S A had RB 2, while 10 had RB 3. The chi-square test showed that 16S and RB phylotypes were not statistically independent of each other within host specimens (*p*-value = 0.00049).

Some patterns in the spatial distribution of symbiont 16S rRNA and RuBisCO phylotypes within Bonne Bay were observed, and chi-square tests revealed that site and phylotype were not statistically independent of each other (*p*-value <0.003 for both genes). Phylotypes 16S A and B were identified at all sampling sites. 16S A was more common than B at Deer Arm and South East Arm, and was rare at Neddy’s Harbour (Fig. 1). Phylotypes 16S C (*N* = 4) and RB 1 (*N* = 2) were found in the same bivalve specimens, all collected in Neddy’s Harbour, suggesting site restriction. RB 3 was found at Deer Arm (*N* = 4) and South East Arm (*N* = 8), but not at Neddy’s Harbour.

Specimens were collected over a span of four years, and hosts with symbiont phylotypes 16S A, 16S B, RB 2 and RB 3 were identified in all months and years (Table S1). Symbiont phylotypes 16S C and RB 1 were only identified in 2010 and 2011. Chi-square tests revealed that sampling date and phylotypes were statistically independent of each other (*p*-values > 0.7 for both genes). We found no apparent correspondence between host OTU and symbiont phylotype; the more common OTU 1 associated with all symbiont phylotypes, and the three individuals of host OTU 2 contained symbionts with 16S A/RB 2, 16S C/RB 1, and 16S C/unknown RB respectively. Finally, host size (Table S1) showed no obvious relationship with symbiont phylotype (chi-square *p*-value > 0.1 for both genes).

**Table 2:** Co-occurrence of 16S rRNA and RuBisCO phylotypes within individual *Thyasira* cf. *gouldi* specimens. Values are numbers of specimens in which each combination of 16S rRNA and RuBisCO phylotype was identified. Only specimens showing no evidence of multiple RuBisCO phylotypes are considered here.

16S Phylotype	RuBisCo Phylotype		
	1	2	3
A	0	6	10
B	0	15	0
C	2	0	0

## Discussion

*Thyasira* cf. *gouldi* hosts a single species of gammaproteobacteria comprising three 16S rRNA subtypes, previously described as phylotypes A, B and C (Batstone & Dufour, 2016). Whereas the previously reported sequences included some degenerate bases, we obtained herein sequences that matched those of Batstone & Dufour (2016) but presented no base ambiguities, and used them as reference sequences (Table S2). The 16S rRNA symbiont diversity observed in the two *T.* cf. *gouldi* OTUs is greater than that found in some lucinid clams (Brissac et al., 2016) and some vestimentiferan tubeworms (Vrijenhoek, Duhaime & Jones, 2007), where a single symbiont phylotype is found in multiple host species. However, evolutionary distances observed between pairs of *T.* cf. *gouldi* symbiont 16S sequences were small (0.002–0.008), with no measurable distance within phylotypes. In contrast, the multiple, co-occurring extracellular symbiont 16S phylotypes observed in some bathymodiolins are more phylogenetically divergent (Duperron et al., 2008a). *T.* cf. *gouldi* therefore associate with a more restricted group of extracellular symbionts than some bathymodiolins, at least with respect to 16S rRNA gene diversity.

A comparably higher degree of variability and evolutionary distance was observed in the *Thyasira* cf. *gouldi* symbiont RuBisCO sequences. Similar RuBisCO polymorphism was observed in symbionts of *Escarpia spicata* and *Lamellibrachia barhami*, but not in those of *Riftia pachyptila* (Vrijenhoek, Duhaime & Jones, 2007). Therefore, in some hosts (including *T*. cf. *gouldi*), the RuBisCO gene is a useful tool for examining symbiont diversity and specificity.

### Symbiont diversity and spatial-temporal patterns

The greater number of samples analyzed herein has led to a revision of the spatial distribution of 16S rRNA phylotypes since Batstone & Dufour (2016), notably through a greater 16S rRNA phylotype diversity at Deer Arm and South East Arm than previously reported. It is now apparent that 16S A and 16S B are widespread in Bonne Bay, while 16S C has only been identified in Neddy’s Harbour. Some RuBisCO phylotypes occurred at multiple sampling sites, but RB1, found only in hosts having 16S C, appeared restricted to the shallower Neddy’s Harbour site. The restriction of 16S C/RB 1 to a single site supports the symbiont environmental acquisition mode proposed for thyasirids (Duperron et al., 2012; Dufour et al., 2014).

The ecotype hypothesis proposes that bacterial strains assemble in relation to environmental factors such as sedimentary organic matter, grain size, and sulfur content. Therefore, in environmentally acquired symbionts, the distribution of symbiont phylotypes will reflect these habitat characteristics (Brissac et al., 2016). This hypothesis may explain the apparent absence of RB 3, and the possible restriction of the 16S C/ RB 1 phylotype in Neddy’s Harbour, which is the shallowest sampling site, with the lowest organic matter content and coarsest sediments (Batstone & Dufour, 2016). The Deer Arm and South East Arm sites have similar water depths and sediment characteristics (Batstone & Dufour, 2016) that may be conducive to all phylotypes but 16S C/ RB 1. The more widespread RB 2 phylotype may be able to function within a broader range of environmental conditions than RB 1 and RB 3. Environmental patchiness on mm- to cm-scales can explain why thyasirids housing different symbiont phylotypes were found within the same Peterson grab sample (Table S1).

### Symbiont evolution and the relationship between 16S and RuBisCO phylotypes

The *Thyasira* cf. *gouldi* symbiont 16S rRNA and RuBisCO phylogenies are not entirely congruent, although some conservation of gene pairs was identified (Table 2). Notably, while the three symbiont 16S phylotypes are closely related and form a single cluster, RuBisCO genes within the same symbionts are more evolutionarily diverged: RB 1 and RB 2 cluster together and with other symbionts, while RB 3 clusters with free-living bacteria (Fig. 3). A similar pattern in associated 16S rRNA and RuBisCO gene sequences has been noted in autotrophic proteobacteria and cyanobacteria, and attributed to horizontal transfer of the RuBisCO gene amongst phylogenetic lineages (Delwiche & Palmer, 1996; Elsaied & Naganuma, 2001; Kleiner, Petersen & Dubilier, 2012). During their free-living existence, symbionts are exposed to other bacteria in the sediment could undergo horizontal gene transfer events (Dahlberg, Bergström & Hermansson, 1998; Davison, 1999).

While the slight variation in 16S rRNA genes amongst *Thyasira* cf. *gouldi* symbionts may not be reflective of physiological differences, RuBisCO gene variants may be biologically significant, providing fitness benefits to symbionts under particular environmental conditions. The horizontal transfer of genes in bacterial symbionts may increase their metabolic efficiency, and therefore thyasirid symbioses may be particularly flexible by acquiring symbionts that are locally adapted to their microenvironment. *Bathymodiolus septemdierum*, another bivalve with environmentally acquired sulfur oxidizing symbionts, shows symbiont genomic variation linked with differences in metabolic capabilities, thought to be the result of horizontal gene transfer (Ikuta et al., 2016).

### Host-symbiont interaction

As observed previously, the *Thyasira* cf. *gouldi* OTUs present in Bonne Bay do not show co-speciation with their symbionts; rather, both host OTUs 1 and 2 can form symbioses with a restricted, but diverse group of bacteria present in the environment (Batstone & Dufour, 2016).

Further, the identity and position of double-peaks in our sequence alignments suggest that some *T.* cf. *gouldi* individuals may host heterogeneous symbiont populations (i.e., they may show multi-infection, or have mixed symbiont populations, either consisting of multiple strains within a phylotype, or multiple phylotypes; as in (Vrijenhoek, Duhaime & Jones, 2007). Therefore, the symbiont population of thyasirids may resemble that observed in some mytilid species with extracellular, and some with intracellular symbionts (Won et al., 2003; Ikuta et al., 2016), as opposed to the clonal symbiont populations found in lucinids (Brissac, Merçot & Gros, 2011; Brissac et al., 2016).

In bivalves that acquire symbionts from their environment, selectivity may take place as gills undergo development. In lucinids, aposymbiotic juveniles may pick up multiple symbiont strains, which are maintained in undifferentiated cells dispersed throughout the lateral zone of gill filaments (Brissac, Merçot & Gros, 2011). These gill cells later differentiate into mature bacteriocytes (Gros, Frenkiel & Mouëza, 1997). Theoretically, from this mixed infection, the bacteriocytes with the best energetic yield are kept and imprinted with that bacterial strain throughout their lifetime, coupling the adult host with a specific strain of symbiont (Gros et al., 2012; Brissac et al., 2016). In contrast, some *Thyasira* cf. *gouldi* specimens appear to be co-infected by multiple symbiont strains, even as adults (shell sizes of hosts with degenerate bases in the sequence are not smaller than those of hosts having no sequence ambiguity; Table S1). Thyasirids may be capable of acquiring new and genetically mixed symbionts over their lifetime, potentially increasing metabolic fitness of the holobiont in changing environments. The burrowing behaviour of thyasirids, combined with symbiont uptake on the mucociliary surface of the foot with subsequent transfer to gills, is a possible mechanism for the acquisition of new symbionts over the course of the host’s life (Dufour et al., 2014; Zanzerl & Dufour, 2017).

This work highlights the importance of looking past 16S rRNA diversity when investigating symbiont populations, and suggests a greater degree of extracellular symbiont diversity in thyasirids than previously recognized. The ability of *Thyasira* cf. *gouldi* to associate with different symbiont strains may lead to improved fitness within environmentally variable habitats and contribute to its phylogenetic diversity. Further work should examine differences between *T.* cf. *gouldi* symbiont phylotypes and symbiont population heterogeneity in greater detail, through use of clone libraries, genomic, or proteomic investigations (e.g., cloning, next-generation sequencing, *in situ* hybridization). Similar work in other hosts with extracellular symbionts would also be warranted for a more comprehensive understanding of specificity and selectivity in chemosynthetic symbioses.

##  Supplemental Information

10.7717/peerj.3597/supp-1 Figure S1 Chromatographs supporting a heterogeneous symbiont population Ambiguous IUPAC base calls supported by sequencing in both forward and reverse directions, as in A and B, were considered to represent heterogeneous symbiont populations within a host. The pair of forward and reverse chromatographs in C shows a case where heterogeneity was not called (multiple calls were only unambiguous in one direction). ****

10.7717/peerj.3597/supp-2 Figure S2 16S rRNA Maximum Likelihood phylogeny of symbionts of *Thyasira* cf *gouldi* Evolutionary history was inferred using the Hasegawa-Kishino-Yano model (Hasegawa, Kishino & Yano, 1985). The percentage of trees in which taxa clustered as shown is next to the branches, with a log likelihood of −1933.4264. The scale bar represents the number of nucleotide substitutions per site. A total of 1324 nucleotide positions were used in the final dataset. Alignment and tree construction performed using MEGA 7 (Kumar, Stecher & Tamura, 2016).

10.7717/peerj.3597/supp-3 Figure S3 Maximum Likelihood phylogenetic tree of individual RuBisCO sequences with no ambiguities This tree was constructed using the Tamura 3-parameter model (Tamura, 1992). The log likelihood of this tree is −964.1913. 1000 bootstraps were run and bootstrap values are presented next to nodes. A discrete Gamma distribution of 5 categories was used, with the rate variation model allowing evolutionary rate differences between sites. 353 nucleotide positions were used in the final dataset. The scale bar represents the number of nucleotide substitutions per site. Alignment and tree construction performed using MEGA7 (Kumar, Stecher & Tamura, 2016).
**Tamura K. 1992.** Estimation of the number of nucleotide substitutions when there are strong transition-transversion and G+C-content biases. *Molecular Biology and Evolution* **9**:678–687.

10.7717/peerj.3597/supp-4 Table S1 Specimen data Month and year of collection are included in sample names.****Coordinates and depth of sampling sites, and host information (shell width and OTU) are indicated, where available, along with symbiont 16S and RB phylotypes, from this study and Batstone & Dufour (2016).

10.7717/peerj.3597/supp-5 Table S2 GenBank accession numbers of sequences included in the phylogenetic trees

10.7717/peerj.3597/supp-6 Table S3 RuBisCO sequence alignment Only positions that are variable are shown. Coloured bases indicate IUPAC codes that represent 2 or more bases.

10.7717/peerj.3597/supp-7 Supplemental Information 1 Edited 16S sequences used to construct phylogenetic trees

10.7717/peerj.3597/supp-8 Supplemental Information 2 Edited RuBisCo sequences used to construct phylogenetic trees

## Additional Information and Declarations

### Field Study Permissions

The following information was supplied relating to field study approvals (i.e., approving body and any reference numbers): Permits for field sampling were obtained from Fisheries and Oceans Canada.

### Data Availability

The following information was supplied regarding data availability: The raw data has been submitted as Supplementary Files.

## References

[ref-1] Batstone RT, Dufour SC (2016). Closely related thyasirid bivalves associate with multiple symbiont phylotypes. Marine Ecology.

[ref-2] Batstone RT, Laurich JR, Salvo F, Dufour SC (2014). Divergent chemosymbiosis-related characters in *Thyasira* cf. *gouldi* (Bivalvia: Thyasiridae). PLOS ONE.

[ref-3] Blazejak A, Kuever J, Erséus C, Amann R, Dubilier N (2006). Phylogeny of 16S rRNA, ribulose 1, 5-bisphosphate carboxylase/oxygenase, and adenosine 5′-phosphosulfate reductase genes from gamma-and alphaproteobacterial symbionts in gutless marine worms (Oligochaeta) from Bermuda and the Bahamas. Applied and Environmental Microbiology.

[ref-4] Bright M, Bulgheresi S (2010). A complex journey: transmission of microbial symbionts. Nature Reviews Microbiology.

[ref-5] Brissac T, Higuet D, Gros O, Merçot H (2016). Unexpected structured intraspecific diversity of thioautotrophic bacterial gill endosymbionts within the Lucinidae (Mollusca: Bivalvia). Marine Biology.

[ref-6] Brissac T, Merçot H, Gros O (2011). Lucinidae/sulfur-oxidizing bacteria: ancestral heritage or opportunistic association? Further insights from the Bohol Sea (the Philippines). FEMS Microbial Ecology.

[ref-7] Caro A, Gros O, Got P, De Wit R, Troussellier M (2007). Characterization of the population of the sulfur-oxidizing symbiont of *Codakia orbicularis* (Bivalvia, Lucinidae) by single-cell analysis. Applied and Environmantal Microbiology.

[ref-8] Cavanaugh C, McKiness ZP, Newton ILG, Stewart F, Dworkin M, Falkow SI, Rosenberg E, Schleifer K-H, Stackebrandt E (2006). Marine chemosynthetic symbioses. The Prokaryotes.

[ref-9] Dahlberg C, Bergström M, Hermansson M (1998). *In situ* detection of high levels of horizontal plasmid transfer in marine bacterial communities. Applied and Environmental Microbiology.

[ref-10] Dando P, Southward A, Southward E (2004). Rates of sediment sulphide oxidation by the bivalve mollusc *Thyasira sarsi*. Marine Ecology Progress Series.

[ref-11] Davison J (1999). Genetic exchange between bacteria in the environment. Plasmid.

[ref-12] Delwiche CF, Palmer JD (1996). Rampant horizontal transfer and duplication of RuBisCO genes in eubacteria and plastids. Molecular Biology and Evolution.

[ref-13] Dubilier N, Bergin C, Lott C (2008). Symbiotic diversity in marine animals: the art of harnessing chemosynthesis. Nature Reviews Microbiology.

[ref-14] Dufour SC (2005). Gill anatomy and the evolution of symbiosis in the bivalve family Thyasiridae. Biological Bulletin.

[ref-15] Dufour SC, Felbeck H (2003). Sulphide mining by the superextensile foot of symbiotic thyasirid bivalves. Nature.

[ref-16] Dufour SC, Laurich JR, Batstone RT, McCuaig B, Elliot A, Poduska KM (2014). Magnetosome-containing bacteria living as symbionts of bivalves. The ISME Journal.

[ref-17] Duperron S, Halary S, Lorion J, Sibuet M, Gaill F (2008a). Unexpected co-occurrence of six bacterial symbionts in the gills of the cold seep mussel *Idas* sp. (Bivalvia: Mytilidae). Environmental Microbiology.

[ref-18] Duperron S, Laurent MCZ, Gaill F, Gros O (2008b). Sulphur-oxidizing extracellular bacteria in the gills of Mytilidae associated with wood falls. FEMS Microbiology Ecology.

[ref-19] Duperron S, Rodrigues CF, Léger N, Szafranski K, Decker C, Olu K, Gaudron SM (2012). Diversity of symbioses between chemosynthetic bacteria and metazoans at the Guiness cold seep site (Gulf of Guinea, West Africa). Microbiology Open.

[ref-20] Elsaied H, Naganuma T (2001). Phylogenetic diversity of ribulose-1, 5-bisphosphate carboxylase/oxygenase large-subunit genes from deep-sea microorganisms. Applied and Environmental Microbiology.

[ref-21] Felbeck H (1981). Chemoautotrophic potential of the hydrothermal vent tube worm, *Riftia pachyptila* Jones (Vestimentifera). Science.

[ref-22] Goffredi S, Hurtado L, Hallam S, Vrijenhoek R (2003). Evolutionary relationships of deep-sea vent and cold seep clams (Mollusca: Vesicomyidae) of the “*pacifica/lepta*” species complex. Marine Biology.

[ref-23] Gros O, Elisabeth NH, Gustave SDD, Caro A, Dubilier N (2012). Plasticity of symbiont acquisition throughout the life cycle of the shallow-water tropical lucinid *Codakia orbiculata* (Mollusca: Bivalvia). Environmental Microbiology.

[ref-24] Gros O, Frenkiel L, Mouëza M (1997). Embryonic, larval, and post-larval development in the symbiotic clam *Codakia orbicularis* (Bivalvia: Lucinidae). Invertebrate Biology.

[ref-25] Hakonen A, Hulth S, Dufour S (2010). Analytical performance during ratiometric long-term imaging of pH in bioturbated sediments. Talanta.

[ref-26] Ihaka R, Gentleman R (1996). R: a language for data analysis and graphics. Journal of Computational and Graphical Statistics.

[ref-27] Ikuta T, Takaki Y, Nagai Y, Shimamura S, Tsuda M, Kawagucci S, Aoki Y, Inoue K, Teruya M, Satou K, Teruya K, Shimoji M, Tamotsu H, Hirano T, Maruyama T, Yoshida T (2016). Heterogeneous composition of key metabolic gene clusters in a vent mussel symbiont population. The ISME Journal.

[ref-28] Jones ML (1981). *Riftia pachyptila* Jones: observations on the vestimentiferan worm from the Galapagos Rift. Science.

[ref-29] Kleiner M, Petersen JM, Dubilier N (2012). Convergent and divergent evolution of metabolism in sulfur-oxidizing symbionts and the role of horizontal gene transfer. Current Opinion in Microbiology.

[ref-30] Kumar S, Stecher G, Tamura K (2016). MEGA7: molecular evolutionary genetics analysis version 7.0 for bigger datasets. Molecular Biology and Evolution.

[ref-31] Lane DJ, Stackebrandt E, Goodfellow M (1991). 16S/23S rRNA sequencing. Nucleic Acid Techniques in Bacterial Systematics.

[ref-32] McFall-Ngai M, Hadfield MG, Bosch TC, Carey HV, Domazet-Lošo T, Douglas AE, Dubilier N, Eberl G, Fukami T, Gilbert SF (2013). Animals in a bacterial world, a new imperative for the life sciences. Proceedings of the National Academy of Sciences of the United States of America.

[ref-33] Moran NM, McCutcheon JP, Nakabachi A (2008). Genomics and evolution of heritable bacterial symbionts. Annual Review of Genetics.

[ref-34] Nei M, Kumar S (2000). Molecular evolution and phylogenetics.

[ref-35] Nussbaumer AD, Fisher CR, Bright M (2006). Horizontal endosymbiont transmission in hydrothermal vent tubeworms. Nature.

[ref-36] Ott JA, Novak R, Schiemer F, Hentschel U, Nebelsick M, Polz M (1991). Tackling the sulfide gradient—a novel strategy involving marine nematodes and chemolithotrophic ectosymbionts. Marine Ecology.

[ref-37] Petersen JM, Wentrup C, Verna C, Knittel K, Dubilier N (2012). Origins and evolutionary flexibility evolutionary flexibility of chemosynthetic symbionts from deep-sea animals. The Biological Bulletin.

[ref-38] Rodrigues CF, Duperron S (2011). Distinct symbiont lineages in three thyasirid species (Bivalvia: Thyasiridae) from the eastern Atlantic and Mediterranean Sea. Die Naturwissenschaften.

[ref-39] Sachs JL, Skophammer RG, Regus JU (2011). Evolutionary transitions in bacterial symbiosis. Proceedings of the National Academy of Sciences of the United States of America.

[ref-40] Tamura K, Nei M (1993). Estimation of the number of nucleotide substitutions in the control region of mitochondrial DNA in humans and chimpanzees. Molecular Biology and Evolution.

[ref-41] Thompson JD, Higgins DG, Gibson TJ (1994). CLUSTAL W: improving the sensitivity of progressive multiple sequence alignment through sequence weighting, position-specific gap penalties and weight matrix choice. Nucleic Acids Research.

[ref-42] Vrijenhoek RC, Kiel S (2010). Genetics and evolution of deep-sea chemosynthetic bacteria and their invertebrate hosts. The vent and seep biota.

[ref-43] Vrijenhoek RC, Duhaime M, Jones WJ (2007). Subtype variation among bacterial endosymbionts of tubeworms (Annelida: Siboglinidae) from the Gulf of California. The Biological Bulletin.

[ref-44] Wernegreen JJ (2005). For better or worse: genomic consequences of intracellular mutualism and parasitism. Current Opinion in Genetics & Development.

[ref-45] Widmer F, Seidler RJ, Gillevet PM, Watrud LS, Di Giovanni GD (1998). A highly selective PCR protocol for detecting 16S rRNA genes of the genus *Pseudomonas* (sensu stricto) in environmental samples. Applied and Environmental Microbiology.

[ref-46] Won YJ, Hallam SJ, O’Mullan GD, Pan IL, Buck KR, Vrijenhoek RC (2003). Environmental acquisition of thiotrophic endosymbionts by deep-sea mussels of the genus *Bathymodiolus*. Applied and Environmental Microbiology.

[ref-47] Won YJ, Jones WJ, Vrijenhoek RC (2008). Absence of cospeciation between deep-sea mytilids and their thiotrophic endosymbionts. Journal of Shellfish Research.

[ref-48] Zanzerl H, Dufour SC (2017). The burrowing behaviour of symbiotic and asymbiotic thyasirid bivalves. Journal of Conchology.

[ref-49] Zimmerman J, Lott C, Weber M, Ramette A, Bright M, Dubilier N, Petersen JM (2014). Dual symbiosis with co-occurring sulfur-oxidizing symbionts in vestimentiferan tubeworms from a Mediterranean hydrothermal vent. Environmental Microbiology.

