# Supplementary figures and images for: The bivalve Thyasira cf. gouldi hosts chemoautotrophic symbiont populations with strain level diversity
Source: PeerJ. 2017 Jul 26;5:e3597. doi: 10.7717/peerj.3597 (PMC5533157; doi:10.7717/peerj.3597)

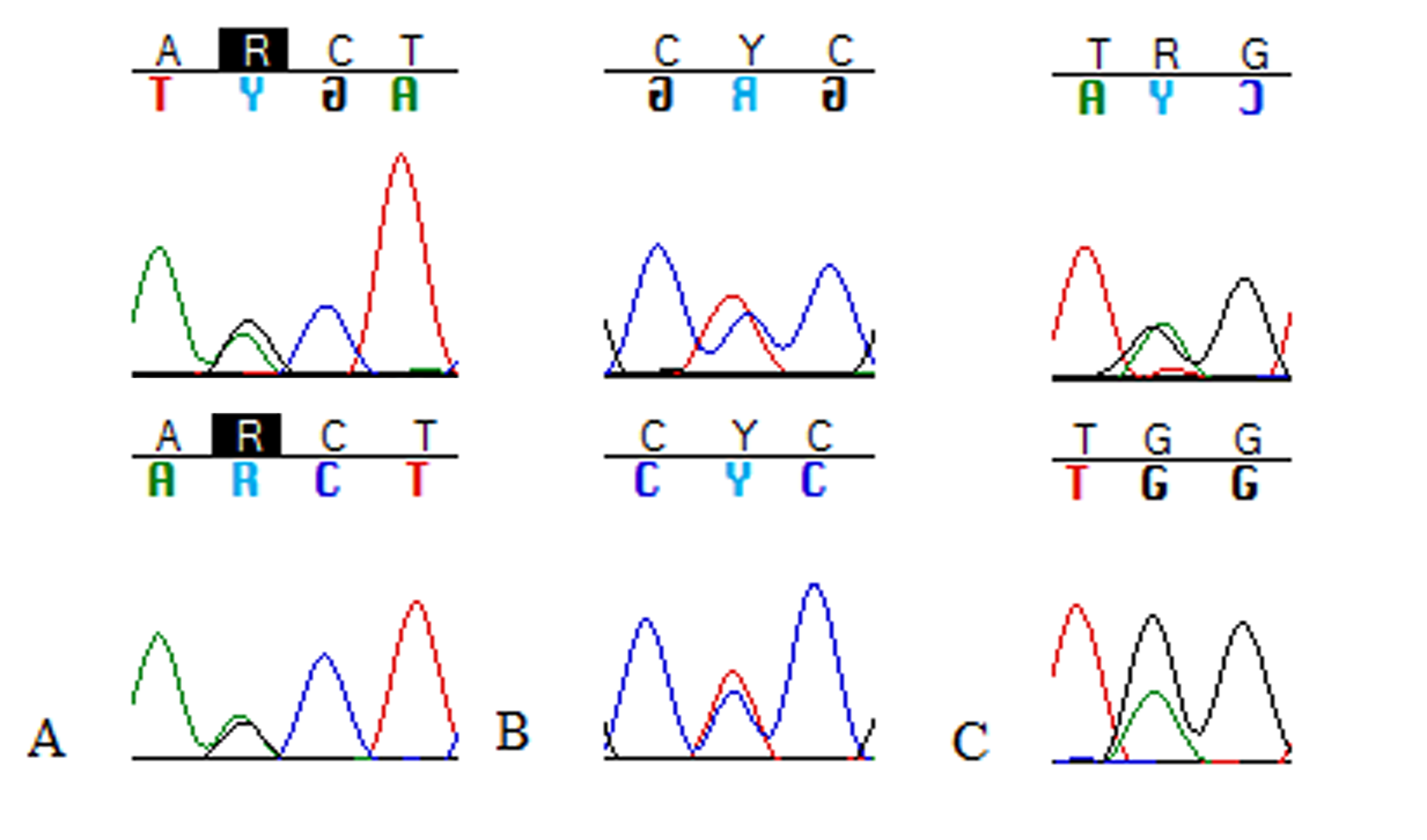

Supplement: Figure S1 — Ambiguous IUPAC base calls supported by sequencing in both forward and reverse directions, as in A and B, were considered to represent heterogeneous symbiont populations within a host. The pair of forward and reverse chromatographs in C shows a case where heterogeneity was not called (multiple calls were only unambiguous in one direction). [file peerj-05-3597-s001.png]

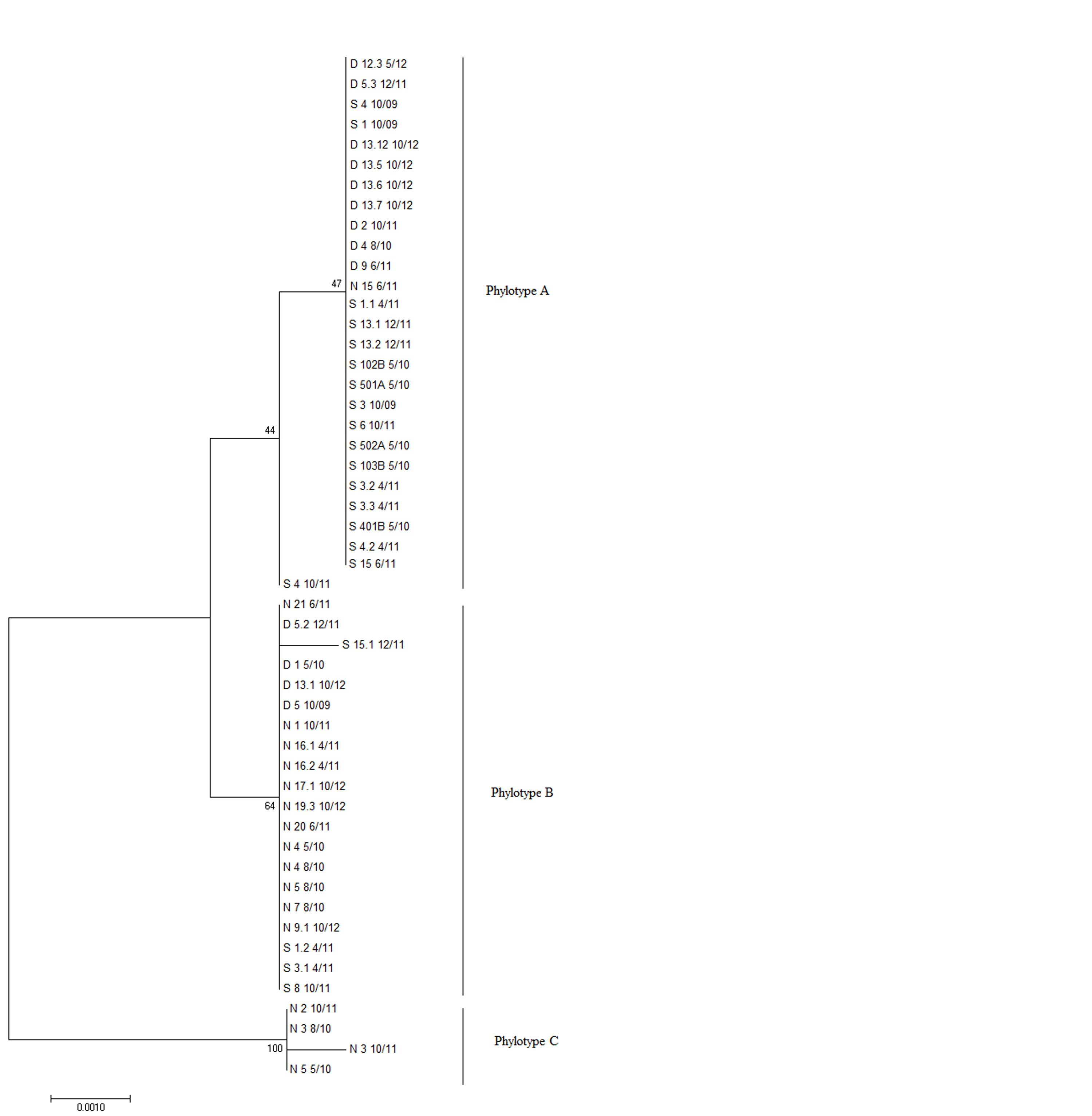

Supplement: Figure S2 — Evolutionary history was inferred using the Hasegawa-Kishino-Yano model (Hasegawa, Kishino & Yano, 1985). The percentage of trees in which taxa clustered as shown is next to the branches, with a log likelihood of −1933.4264. The scale bar represents the number of nucleotide substitutions per site. A total of 1324 nucleotide positions were used in the final dataset. Alignment and tree construction performed using MEGA 7 (Kumar, Stecher & Tamura, 2016). [file peerj-05-3597-s002.png]

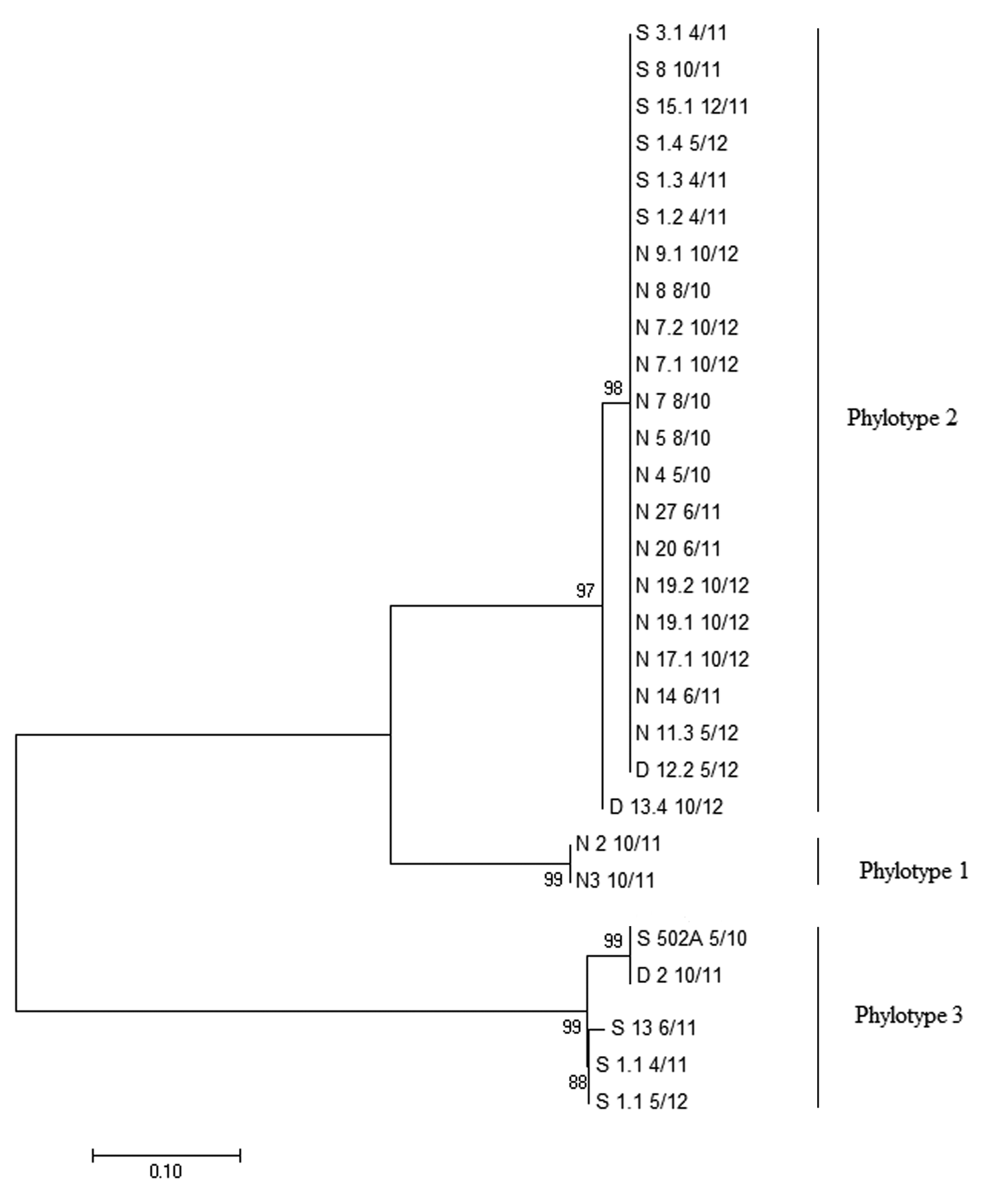

Supplement: Figure S3 — This tree was constructed using the Tamura 3-parameter model (Tamura, 1992). The log likelihood of this tree is −964.1913. 1000 bootstraps were run and bootstrap values are presented next to nodes. A discrete Gamma distribution of 5 categories was used, with the rate variation model allowing evolutionary rate differences between sites. 353 nucleotide positions were used in the final dataset. The scale bar represents the number of nucleotide substitutions per site. Alignment and tree construction performed using MEGA7 (Kumar, Stecher & Tamura, 2016). Tamura K. 1992. Estimation of the number of nucleotide substitutions when there are strong transition-transversion and G+C-content biases. Molecular Biology and Evolution 9:678–687. [file peerj-05-3597-s003.png]
