# Supplementary material for: The bivalve Thyasira cf. gouldi hosts chemoautotrophic symbiont populations with strain level diversity
Source: PeerJ. 2017 Jul 26;5:e3597. doi: 10.7717/peerj.3597 (PMC5533157; doi:10.7717/peerj.3597)
Supplement: Table S1 — Month and year of collection are included in sample names.Coordinates and depth of sampling sites, and host information (shell width and OTU) are indicated, where available, along with symbiont 16S and RB phylotypes, from this study and Batstone & Dufour (2016). [file peerj-05-3597-s004.docx]

| **Sample ID**  **mm/yy** | **Coordinates** | | **Depth (m)** | **Shell width (mm)** | **Host OTU** | **16S** | **RB** |
| --- | --- | --- | --- | --- | --- | --- | --- |
|  | **N** | **W** |  |  |  |  |  |
| **Deer Arm** | | | | | | | |
| D 5 10/09 | - | - | - | - | - | B | - |
| D 1 5/10 | 49˚33.236 | 57˚50.377 | - | 3 | 1 | B | - |
| D 4 8/10 | - | - | - | 2 | - | A | - |
| D 6 6/11 | - | - | - | 3.2 | 1 | - | Mixed |
| D 9 6/11 | - | - | - | 3.2 | 1 | A | - |
| D 2 10/11 | 49°33.222 | 57°50.445 | 31 | 3.5 | - | A | 3 |
| D 5.2 12/11 | 49°33.219 | 57°50.384 | 34 | 4 | - | B | 2 |
| D 5.3 12/11 | 9°33.219 | 57°50.380 | 34 | 4.7 | - | A | - |
| D 8.1 5/12 | 49°33.236 | 57°50.409 | 29 | 2.4 | - | - | 2 |
| D 12.2 5/12 | 49°33.201 | 57°50.431 | 36 | 3.2 | - | - | 2 |
| D 12.3 5/12 | 49°33.201 | 57°50.431 | 36 | 4.2 | - | A | 3 |
| D 13.1 10/12 | 49°33.210 | 57°50.420 | 34 | 4 | - | B | - |
| D 13.2 10.12 | 49°33.210 | 57°50.420 | 34 | 5 | - | - | Mixed |
| D 13.4 10/12 | 49°33.210 | 57°50.420 | 34 | 3.1 | - | - | 2 |
| D 13.5 10/12 | 49°33.210 | 57°50.420 | 34 | 4.3 | - | A | 2 |
| D 13.6 10/12 | 49°33.210 | 57°50.420 | 34 | 4.2 | - | A | 3 |
| D 13.7 10/12 | 49°33.210 | 57°50.420 | 34 | 3.9 | - | A | - |
| D 13.12 10/12 | 49°33.210 | 57°50.420 | 34 | 3.5 | - | A | 3 |
| **Neddy's Harbour** | | | | | | | |
| N 4 5/10 | 49˚27.372 | 57˚53.280 | 20 | - | 1 | B | 2 |
| N 5 5/10 | 49˚27.372 | 57˚53.280 | 20 | - | 1 | C | - |
| N 3 8/10 | - | - | - | - | 2 | C | - |
| N 4 8/10 | - | - | - | - | 1 | B | - |
| N 5 8/10 | - | - | - | 2.5 | 1 | B | 2 |
| N 7 8/10 | - | - | - | 2.5 | 1 | B | 2 |
| N 8 8/10* | - | - | - | 3.5 | 1 | B | 2 |
| N 16.1 4/11 | 49°31.460 | 57°52.230 | 17 | 2.2 | 1 | B | 2 |
| N 16.2 4/11 | 49°31.460 | 57°52.230 | 17 | 2.8 | 1 | B | - |
| N 14 6/11 | - | - | - | 2.3 | 1 | - | 2 |
| N 15 6/11 | - | - | - | 3 | - | A | 2 |
| N 20 6/11 | - | - | - | 3.1 | 1 | B | 2 |
| N 21 6/11 | - | - | - | 3.2 | 1 | B | 2 |
| N 27 6/11 | - | - | - | 2.7 | 1 | - | 2 |
| N 1 10/11 | - | - | - | 3 | 1 | B | - |
| N 2 10/11 | - | - | - | 3.2 | - | C | 1 |
| N 3 10/11 | - | - | - | 2.5 | 2 | C | 1 |
| N 11.3 5/12 | 49°31.386 | 57°52.186 | 30 | 3 | - | - | 2 |
| N 7.1 10/12 | 49°31.436 | 57°52.234 | - | 2.5 | - | - | 2 |
| N 7.2 10/12 | 49°31.436 | 57°52.234 | - | 2.3 | - | - | 2 |
| N 9.1 10/12 | 49°31.431 | 57°52.275 | - | 2.5 | - | B | 2 |
| N 17.1 10/12 | 49°31.456 | 57°52.246 | - | 2.7 | - | B | 2 |
| N 19.1 10/12 | 49°31.420 | 57°52.240 | - | 2.6 | - | - | 2 |
| N 19.2 10/12 | 49°31.420 | 57°52.240 | - | 2.6 | - | - | 2 |
| N 19.3 10/12 | 49°31.420 | 57°52.240 | - | 2.5 | - | B | 2 |
| **South East Arm** | | | | | | | |
| S 1 10/09 | - | - | - | - | - | A | - |
| S 3 10/09 | - | - | - | - | - | A | - |
| S 4 10/09 | - | - | - | - | - | A | - |
| S 102B 5/10 | 49˚27.774 | 57˚43.493 | 30 | - | 2 | A | 3 |
| S 103B 5/10 | 49˚27.774 | 57˚43.493 | 30 | - | 1 | A | 2 |
| S 401B 5/10 | 49˚27.723 | 57˚43.455 | 20 | 5 | 1 | A | - |
| S 501A 5/10 | 49˚27.720 | 57˚43.466 | 20 | - | 1 | A | 2 |
| S 502A 5/10 | 49˚27.720 | 57˚43.466 | 20 | - | 1 | A | 3 |
| S 1.1 4/11 | 49°27.748 | 57°42.773 | 27 | 4 | 1 | A | 3 |
| S 1.2 4/11 | 49°27.748 | 57°42.773 | 27 | 3 | 1 | B | 2 |
| S 1.3 4/11 | 49°27.752 | 57°42.449 | 25 | 3.5 | 1 | - | 2 |
| S 3.1 4/11 | 49°27.751 | 57°42.822 | 30 | 4 | 1 | B | 2 |
| S 3.2 4/11 | 49°27.751 | 57°42.822 | 30 | 3 | 1 | A | - |
| S 3.3 4/11 | 49°27.751 | 57°42.822 | 30 | 3.5 | - | A | 2 |
| S 4.2 4/11 | 49°27.745 | 57°42.806 | 30 | 3.5 | - | A | - |
| S 12 6/11* | - | - | - | 2.5 | 1 | A | 3 |
| S 13 6/11* | - | - | - | 4 | 1 | A | 3 |
| S 15 6/11 | - | - | - | 2.7 | - | A | Mixed |
| S 4 10/11 | - | - | - | 4 | - | A | 2 |
| S 6 10/11 | - | - | - | 3.8 | - | A | - |
| S 8 10/11 | - | - | - | 3.2 | 1 | B | 2 |
| S 13.1 12/11 | 49°27.787 | 57°42.482 | 35 | - | 1 | A | - |
| S 13.2 12/11 | 49°27.787 | 57°42.482 | 35 | - | - | A | 3 |
| S 15.1 12/11 | - | - | - | 4.2 | 1 | B | 2 |
| S 1.1 5/12 | 49°27.837 | 57°42.886 | 31 | 5 | - | - | 3 |
| S 1.4 5/12 | 49°27.837 | 57°42.886 | 31 | 3.3 | - | - | 2 |
| S 6.4 5/12 | 49°27.827 | 57°42.852 | 30 | 4 | - | - | 3 |

* 16S rRNA sequences unavailable for use in genetic trees
