# Supplementary material for: The bivalve Thyasira cf. gouldi hosts chemoautotrophic symbiont populations with strain level diversity
Source: PeerJ. 2017 Jul 26;5:e3597. doi: 10.7717/peerj.3597 (PMC5533157; doi:10.7717/peerj.3597)
Supplement: Table S2 [file peerj-05-3597-s005.docx]

| Organism Name | 16S rRNA  Accession number | RuBisCO  Accession Number |
| --- | --- | --- |
| *Thyasira* cf. *gouldi* symbiont phylotype A | MF040754 |  |
| *Thyasira* cf. *gouldi* symbiont phylotype B | MF040755 |  |
| *Thyasira* cf. *gouldi* symbiont phylotype C | MF040756 |  |
| *Thyasira* cf. *gouldi* symbiont phylotype 1 |  | MF040758 |
| *Thyasira* cf. *gouldi* symbiont phylotype 2 |  | MF040759 |
| *Thyasira* cf. *gouldi* symbiont phylotype 3 |  | MF040757 |
| *Calyptogena okutanii* symbiont | AP009247.1 | AP009247.1 |
| *Acharax* sp. *Guiness* symbiont | HE863797.1 | HE863799.1 |
| *Lamellibrachia* sp. symbiont | FM165437.1 | FM165442.1 |
| *Sedimenticola thiotaurini* strain SIP-G1 | CP011412.1 | CP011412.1 |
| *Riftia pachyptila* (vent Ph05) symbiont | AY129116.2 | AF047688.1 |
| *Halothiobacillus* sp. LS2 | CP016027.1 | CP016027.1 |
| *Thioflavicoccus mobilis* 8321 | CP003051.1 | CP003051.1 |
| *Thiomonas* sp. CB2 | LK931616.1 | LK931649.1 |
| *Thiomonas intermedia* K12 | CP002021.1 | CP002021.1 |
| *Sclerolinum contortum* symbiont | AM883183.1 | AM883192.1 |
| *Thiohalomonas denitrificans* strain HLD3 | EF117910.1 | GQ888609.1 |
| *Thiorhodococcus drewsii* AZ1 | NR 116895.1 | AFWT01000004.1 |
| *Thiorhodovibrio* sp. 970 | FJ815159.1 | JH603168.1 |
| *Oligobrachia haakonmosbiensis* symbiont | HQ877092.1 | HQ877086.1 |
| *Rimicans exoculata* symbiont | FN658699.1 | JH603168.1 |
| *Dechloromonas aromatica* RCB | CP000089.1 | CP000089.1 |
| *Polaromonas naphthalenivorans* CJ2 | CP000529.1 | NC 008781.1 |
| *Leptothrix cholodnii* SP-6 | NC 010524.1 | NC 010524.1 |
| *Paracatenula* sp. symbiont | HQ845110.1 | HQ840958.1 |
| *Magnetospirillium magnetotacticum* | AB680822.1 | AY450592.1 |
| *Phaeospirillium fulvum* strain 5K | HQ877092.1 | HQ877086.1 |
